# A Feasibility Study of a Novel Delayed Cord Clamping Cart

**DOI:** 10.3390/children8050357

**Published:** 2021-04-29

**Authors:** Neha S. Joshi, Kimber Padua, Jules Sherman, Douglas Schwandt, Lillian Sie, Arun Gupta, Louis P. Halamek, Henry C. Lee

**Affiliations:** 1 Department of Pediatrics, Stanford University, Stanford, CA 94305, USA; kpadua@stanford.edu (K.P.); doug.schwandt@gmail.com (D.S.); lillsie@stanford.edu (L.S.); agup@stanford.edu (A.G.); halamek@stanford.edu (L.P.H.); 2 The Hasso Plattner Institute of Design, Stanford University, Stanford, CA 94305, USA; julessherman@alumni.stanford.edu

**Keywords:** neonatology, resuscitation, delayed cord clamping, simulation

## Abstract

Delaying umbilical cord clamping (DCC) for 1 min or longer following a neonate’s birth has now been recommended for preterm and term newborns by multiple professional organizations. DCC has been shown to decrease rates of iron deficiency anemia, intraventricular hemorrhage (IVH), necrotizing enterocolitis (NEC), and blood transfusion. Despite these benefits, clinicians typically cut the umbilical cord without delay in neonates requiring resuscitation and move them to a radiant warmer for further care; this effectively prevents these patients from receiving any benefits from DCC. This study evaluated the feasibility of a delayed cord clamping cart (DCCC) in low-risk neonates born via Cesarean section (CS). The DCCC is a small, sterile cart designed to facilitate neonatal resuscitation while the umbilical cord remains intact. The cart is cantilevered over the operating room (OR) table during a CS, allowing the patient to be placed onto it immediately after birth. For this study, a sample of 20 low-risk CS cases were chosen from the non-emergency Labor and Delivery surgical case list. The DCCC was utilized for 1 min of DCC in all neonates. The data collected included direct observation by research team members, recorded debriefings and surveys of clinicians as well as surveys of patients. Forty-four care team members participated in written surveys; of these, 16 (36%) were very satisfied, 12 (27%) satisfied, 13 (30%) neutral, and 3 (7%) were somewhat dissatisfied with use of the DCCC in the OR. Feedback was collected from all 20 patients, with 18 (90%) reporting that they felt safe with the device in use. This study provides support that utilizing a DCCC can facilitate DCC with an intact umbilical cord.

## 1. Introduction

Delayed cord clamping (DCC) refers to the practice of delaying clamping of a neonate’s umbilical cord for at least 30–60 s after birth. This delay in clamping of the umbilical cord effectively allows for a blood transfusion from the placental bed into the newborn’s circulation [1,2,3,4,5]. Studies suggest that 75% of available blood in the placenta is transfused to the infant within 1 min [6]. DCC has noted benefits in both preterm and term neonates. In preterm neonates, DCC is associated with increased hematocrit levels, decreased need for blood transfusion, and decreased incidence of NEC and IVH [7,8,9]. Term neonates receiving DCC have increased hemoglobin levels at birth, increased iron stores at 6 months of age, and potentially improved neurodevelopmental outcomes [6,7,8,9,10]. Given its known benefits, DCC has been endorsed by multiple professional organizations, including the World Health Organization, the International Liaison Committee on Resuscitation, the American Academy of Pediatrics, and the American College of Obstetricians and Gynecologists, amongst several others [1,2,3,4,5,11]. These professional organizations recommend DCC in most vigorous preterm and term neonates.

Some reasons for not performing DCC include the need for immediate neonatal resuscitation, maternal bleeding, and concern for an intact maternal-neonatal umbilical cord circulation. Neonates requiring immediate resuscitation often receive immediate cord clamping due to the challenging ergonomics of performing resuscitation during DCC. Following cord clamping, these patients are carried to a radiant warmer provisioned with resuscitation equipment and supplies such as oxygen, suctioning, and positive pressure ventilation capability.

Providing continuous positive airway pressure (CPAP) immediately at birth to premature neonates born at <28 weeks gestational age reduces the need for respiratory interventions such as exogenous surfactant administration and mechanical ventilation while potentially reducing the severity of respiratory distress syndrome and incidence of bronchopulmonary dysplasia [12,13]. Presently, it is not practical in most circumstances for neonates to receive both immediate CPAP or other forms of neonatal resuscitation, and also receive DCC, given the logistical difficulties of current standard hospital equipment.

To address the logistical challenges of providing resuscitation including CPAP or positive pressure ventilation during DCC, our team designed a sterile compact cart (the Delayed Cord Clamping Cart, DCCC) that can be positioned near the incision site during a CS or by a mother’s bedside during a vaginal delivery, allowing for resuscitation to be performed during DCC. We conducted a study in order to evaluate the feasibility of using this DCCC during CS deliveries.

## 2. Materials and Methods

### 2.1. Cart Design

**Figure 1:**
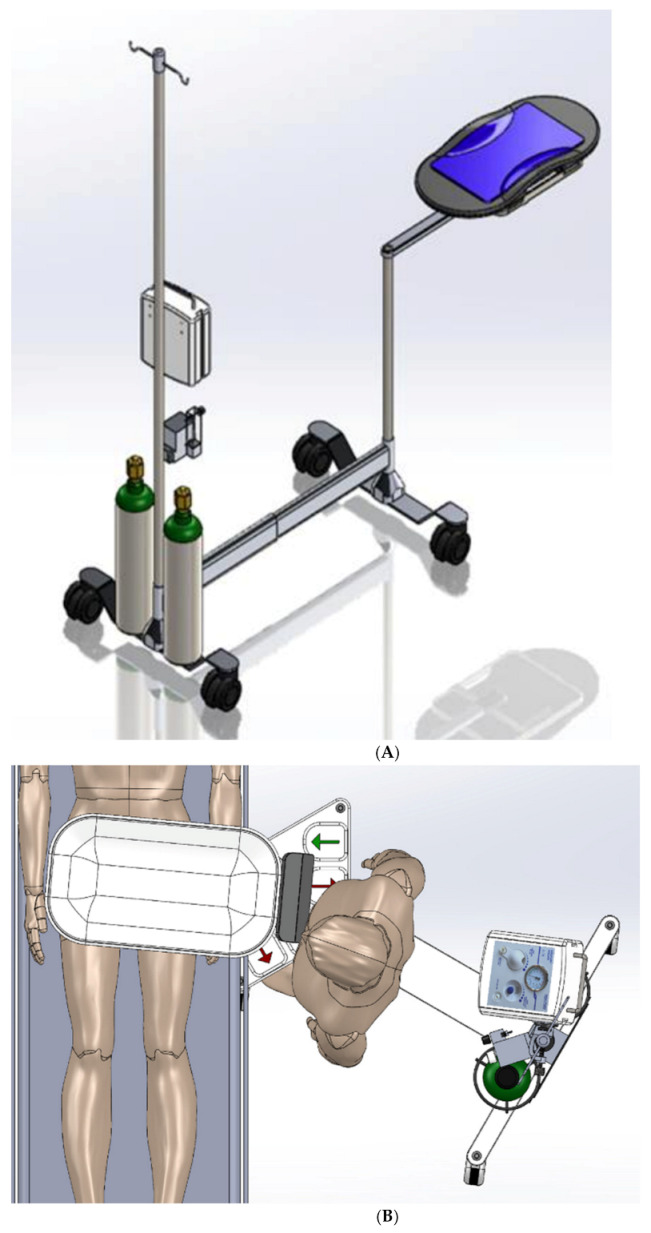
(**A**) Schematic rendering of delayed cord clamping cart. (**B**) Schematic rendering of delayed cord clamping cart cantilevered over patient’s abdomen during CS.

Our research team had previously performed simulations and debriefings of childbirth focusing on DCC while resuscitating the infant [14]. Based on this work, the team designed and prototyped a cart to facilitate DCC while allowing for the possibility of simultaneous respiratory support (Figure 1). Iterations of the prototype occurred based on further repeated simulations involving a multidisciplinary team at the Stanford Medicine Center for Advanced Pediatric and Perinatal Education (CAPE, http://cape.stanford.edu, accessed on 28 April 2021). The development of the DCCC began with simple concept sketches, and subsequently evolved to increasingly more complex functional models using SolidWorks Premium 2021 computer aided design (CAD) software. These models focused on planning the mechanical structure, mechanisms, and electrical features of the cart.

For stability purposes, it was essential for the DCCC cart base to have sufficient floor support in addition to the ballast required to counterweight the extension of the tray well beyond the wheeled base. Initially, simple support blades similar to those found on traditional Mayo stands, were trialed to minimize tipping of the DCCC. These were subsequently updated to utilizing castering wheels, in order to maximize easy mobility of the DCCC. An intravenous pole was placed in the back with the requisite resuscitation equipment. Air and oxygen cylinders were strategically placed at the base of the intravenous pole to help generate sufficient ballast for extending the resuscitation tray forward to allow it to safely cantilever over the operating table. Tipping analyses were performed, taking into account dynamic movement of the cart.

A single telescoping column was placed at the front of the cart to provide approximately 65 cm (25.6 in) of vertical adjustment, allowing the tray to be adjusted from about 68 to 133 cm (26.8 to 52.3 in) height above the floor. The DCCC’s arm extends from a proximal rotary joint at the top of the column to another distal rotary joint below the tray. A prismatic sliding joint under the tray provides additional reach. The telescoping column combined with the rotary and prismatic joints allow for flexibility in both positioning and orientation of the tray to make it easier to safely place the neonate onto the DCCC tray without putting excessive traction on the neonate’s umbilical cord.

A fully functional version of the DCCC incorporating these features was designed and fabricated. Simulations of the DCCC were conducted, with feedback received from the multidisciplinary working group. Once shown to be safe and effective during simulated deliveries, the DCCC was approved for use by the Biomedical Engineering Department at Lucile Packard Children’s Hospital Stanford and this study was authorized by the Institutional Review Board (IRB) of Stanford University.

**Figure 2:**
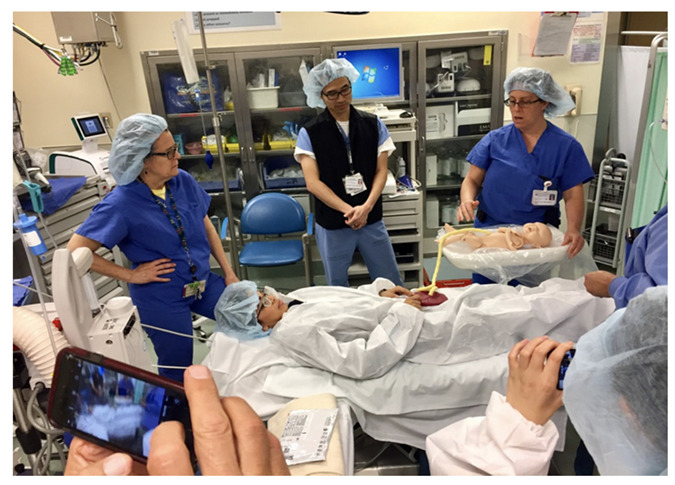
Delayed cord clamping cart in use during a simulation.

The DCCC is a small, mobile sterile cart (Figure 1 and Figure 2) designed to facilitate resuscitation while the umbilical cord is intact. The DCCC is designed to cantilever, extending from the cart over the operating room (OR) table during a CS, allowing for the newborn to be placed directly onto it immediately at the time of birth. As described above, the DCCC is equipped with mechanisms that facilitate vertical movement, positioning and orientation over the OR table, and wheels for maneuverability around the room. It is designed to accommodate the equipment used during neonatal resuscitation.

### 2.2. Study

A sample of 20 scheduled low-risk CS cases were chosen to pilot the DCCC. Written informed consent was obtained from patients and the members of the multidisciplinary OR team (pediatricians, obstetricians, anesthesiologists, nursing staff, and surgical technicians). Each multidisciplinary team member was trained in the use of the DCCC.

In this observational study, a research team member observed the use of the DCCC and its effectiveness in facilitating DCC during CS. Without the DCCC, the standard procedure includes placing the neonate on the mother’s abdomen or having a clinician hold the neonate during DCC. With the DCCC, the neonate was placed directly onto the cart at the time of delivery. Once the cord was clamped, the patient was moved to the radiant warmer while remaining on the DCCC, then transitioned to the radiant warmer for further evaluation and care. Otherwise, the cart did not influence or change the current provider procedures in the operating room. Video recordings of the procedure were made, and structured interviews and written surveys of all team members were conducted after the CS. Written surveys were completed by patients after recovery.

The primary end-point was to evaluate the safety and efficacy of the DCCC. Immediately following each case, the clinicians were asked to participate in a structured interview about their experience using the DCCC and to identify any elements of the cart that could be improved. Patients and their partners were given a short survey 12–24 h postpartum regarding their experience with the DCCC.

### 2.3. Data Analysis

The data presented here reflect approximately 20 h of observation. Demographic data were analyzed using counts and percentages. The structured interviews from patients and providers were transcribed and analyzed for thematic patterns. Once recurring themes from the analyses were identified, the research team made improvements to the cart accordingly. The surveys from the patients were analyzed and triaged for patient safety and patient experience.

## 3. Results

The DCCC was used in 20 CS deliveries of term singletons from October 2018 to January 2020. Gestational age at the time of delivery ranged from 36 to 39 weeks. Enrolled patients were pre-identified as low risk for anticipated maternal and neonatal complications at the time of delivery by the delivering obstetrician. All neonates were vigorous at delivery, and received 60 s of DCC on the DCCC; no neonates required resuscitation.

There were 159 providers who participated in the CS that utilized the DCCC. The multidisciplinary team members directly interacting with the cart included attending neonatal hospitalists, pediatric resident physicians, attending obstetricians, obstetrician resident physicians, and obstetric surgical technicians. Neonatal nurses, obstetric nurses, and members of the anesthesia team were included in the informed consent process but did not directly interact with the cart; formal interviews and written feedback was not collected from those not directly interacting with the cart given that the DCCC neither supported nor impeded their workflow.

Data collected from structured interviews of team members and patients reflects approximately 20 h of recordings, which were transcribed and subsequently analyzed. Forty-four care team members participated in written surveys. Sixteen providers (36%) were very satisfied, 12 (27%) satisfied, 13 (30%) neutral, and 3 (7%) were somewhat dissatisfied with use of the DCCC in the OR. Team members commented that the cart was easily incorporated into the surgical field without concern for breaking sterility, and was not a hindrance during the surgery. An obstetrician noted that the usage of the DCCC “improved baby positioning” compared to without the cart, and another noted that the DCCC “just seems more stable” for the neonate. Given that umbilical cords vary in length, team members noted the need for close evaluation and adjustment in positioning of the DCCC to prevent excessive traction on the cord during DCC. The most frequently noted concern involved maneuvering the cart from the OR table to the radiant warmer. No adverse safety events occurred to either the mother or infant during any of the CS trials with the DCCC.

Feedback was additionally collected from 18 patients, with 2 patients lost to follow up. Seventeen patients (94%) reported that it was easy to communicate with the hospital staff while the device was in use; one patient (5%) felt neutral. Eighteen patients (100%) reported they felt safe when the device was in use. No adverse outcomes for mothers or neonates were noted.

Video recordings of all 20 CS deliveries were watched by the research team. Recurring themes noticed on video recordings and in team member surveys were employed to update the features of the DCCC. These changes included adjustments in the height of the cart’s tray side walls for more secure positioning during the cart’s usage, and adding hand switches under the tray similar to existing foot manual controls to allow for two ways to adjust the DCCC’s height for each delivery.

## 4. Discussion

Facilitating successful DCC has several benefits for both preterm and term neonates. While DCC is now standard of care in vigorous newborns, it is likely that non-vigorous patients requiring resuscitation may also benefit from DCC. Studies in lambs have shown that DCC while the animal establishes ventilation confers hemodynamic stability during the transition to the extrauterine environment [15]. Aeration of the lungs during spontaneous ventilation triggers pulmonary vasodilation and an increase in pulmonary blood flow; this increased pulmonary blood flow then provides critical preload to the left ventricle and supports systemic perfusion and blood pressure [16]. DCC, through its transfusion of placental blood into the neonatal circulation and subsequent increase in left ventricular preload, can thus confer hemodynamic stability to vigorous preterm and term neonates, and may also benefit those who are non-vigorous at birth if they are receiving positive pressure ventilation. Facilitating DCC while allowing for the beginning steps of neonatal resuscitation to take place is especially beneficial in preterm infants, who are at highest need for neonatal resuscitation and also likely stand to benefit the most from DCC’s protective effects.

A mobile cart to facilitate DCC has been previously described in the literature and work continues to refine design and standardize equipment [17,18,19]. There are currently two such carts that are commercially available: the Life Start^®^ Trolley [West Sussex, United Kingdom] and the INSPiRe Platform. However, neither of these devices are specifically designed to get close enough to the mother’s incision site so that a preterm neonate can remain attached to the umbilical cord while CPAP is being performed. In clinical trials, these devices could not be used in 30% of deliveries due to their bulky design [20,21].

We evaluated the ergonomics and feasibility of a sterile DCCC to be utilized during CS and vaginal deliveries in order to enable resuscitation with an intact cord. Given its goals, 20 low-risk CS deliveries in term neonates were selected for inclusion; none of these neonates required resuscitation. The DCCC received overall favorable feedback from team members regarding ease of use. All patients reported that they felt safe during use of the DCCC with their newborns and most noted that they were able to communicate easily with their care team during the DCCC’s usage. Team members often commented on the ease with which the DCCC incorporated into the existing workflow for CS deliveries, which is key to allowing for successful adaptation and implementation of the cart. A limitation of this study is its utilization in only a relatively small number of low-risk CS deliveries of infants not requiring resuscitation; however, data obtained from this study will be used to further refine the DCCC for studies to determine its safety, efficacy, and feasibility in vaginal deliveries and in preterm and term neonates requiring resuscitation at birth. Further studies utilizing the DCCC could additionally capture clinical outcomes of neonatal resuscitation. In summary, our study provides support that the DCCC can facilitate DCC in vigorous term neonates while the umbilical cord remains intact.

## Data Availability

The data presented in this study are available on request from the corresponding author. The data are not publicly available due to privacy concerns.
